# Eureka: objective assessment of the empty pelvis syndrome to measure volumetric changes in pelvic dead space following pelvic exenteration

**DOI:** 10.1007/s10151-024-02952-0

**Published:** 2024-06-26

**Authors:** C. T. West, A. Tiwari, L. Matthews, I. Drami, D. V. C. Mai, J. T. Jenkins, H. Yano, M. A. West, A. H. Mirnezami

**Affiliations:** 1 Southampton Complex Cancer and Exenteration Team, University Hospital Southampton NHS Foundation Trust, Southampton, UK; 2 Cancer Sciences, Faculty of Medicine, University of Southampton, Southampton, UK; 3 St Mark’s Hospital & Academic Institute, London, UK; 4 Imperial College, London, UK

**Keywords:** Pelvic exenteration, Empty pelvis syndrome, Dead space, Volumetrics

## Abstract

**Background:**

Large tissue defects following pelvic exenteration (PE) fill with fluid and small bowel, leading to the empty pelvis syndrome (EPS). EPS causes a constellation of complications including pelvic sepsis and reduced quality of life. EPS remains poorly defined and cannot be objectively measured. Pathophysiology of EPS is multifactorial, with increased pelvic dead space potentially important. This study aims to describe methodology to objectively measure volumetric changes relating to EPS.

**Methods:**

The true pelvis is defined by the pelvic inlet and outlet. Within the true pelvis there is physiological pelvic dead space (PDS) between the peritoneal reflection and the inlet. This dead space is increased following PE and is defined as the exenteration pelvic dead space (EPD). EPD may be reduced with pelvic filling and the volume of filling is defined as the pelvic filling volume (PFV). PDS, EPD, and PFV were measured intraoperatively using a bladder syringe, and Archimedes’ water displacement principle.

**Results:**

A patient undergoing total infralevator PE had a PDS of 50 ml. A rectus flap rendered the pelvic outlet watertight. EPD was then measured as 540 ml. Therefore there was a 10.8-fold increase in true pelvis dead space. An omentoplasty was placed into the EPD, displacing 130 ml; therefore, PFV as a percentage of EPD was 24.1%.

**Conclusions:**

This is the first reported quantitative assessment of pathophysiological volumetric changes of pelvic dead space; these measurements may correlate to severity of EPS. PDS, EPD, and PFV should be amendable to assessment based on perioperative cross-sectional imaging, allowing for potential prediction of EPS-related outcomes.

## Introduction

Pelvic exenteration (PE) constitutes radical surgery for locally advanced and locally recurrent pelvic cancers, and is associated with postoperative complications. The empty pelvis syndrome (EPS) is an emerging concept, occurring in up to 21.9% of cases and estimated to contribute to 40% of the morbidity following PE. EPS is a spectrum of complications manifesting with pelvic sepsis, bowel obstruction, enteric fistulation, and chronic perineal sinus. However, variable definitions and outcomes for EPS in the literature have hindered the identification of effective strategies to mitigate this issue [1, 2]. This is an area of unmet clinical and research need, reflected by the ongoing consensus study by the PelvEx Collaborative [3].

The pathophysiology of EPS is multifactorial and currently principally based on expert opinion. Nevertheless, although inconsistent, the literature describes a large defect, void, vacant space, empty space, dead space, or empty cavity as being implicated [1, 2, 4, 5]. It has been shown that EPS complications are both more likely to occur and require surgical reintervention following more radical PE [1, 6]. Therefore, changes in volumes of pelvic dead space before and after PE may be relevant to the incidence and severity of EPS.

There is currently no methodology described to assess volumetric changes associated with PE. This study aims to demonstrate that alterations in the volumes of pelvic dead space are objectively measurable.

## Materials and methods

The pelvis can be divided into the true (or lesser) pelvis and false pelvis. The true pelvis is defined by the pelvic inlet superiorly and the pelvic outlet inferiorly, with boundaries formed by the pubic bones anteroinferiorly, sacrum and coccyx posteriorly, and the fused ischium and ilium laterally. This space contains the pelvic viscera that may be resected during PE and forms the basis of volumetric assessment. The pelvic inlet is defined by the sacral promontory, pelvic brim, and superior border of the symphysis pubis; the outlet is defined by the ischial tuberosities laterally; anteriorly the inferior ischiopubic rami, and inferior border of the pubic symphysis; and posteriorly the sacrotuberous ligaments and coccyx, and levator ani muscle inferiorly [7].

The true pelvis contains a volume of dead space between the pelvic inlet superiorly and the peritoneal reflection over the rectum inferiorly. This study defines this area as the physiological pelvic dead space (PDS). Following PE and resection of pelvic viscera, the PDS increases, and the inferior border of the pelvic outlet may be disrupted by resection of the levator ani muscle or bony landmarks of the pelvic outlet. Finally, any subsequent reconstruction of the levator ani, for example with simple primary closure, myocutaneous flaps or meshes, will then create a neo-pelvic outlet. The exenteration pelvic dead space (EPD) is therefore defined here as the volume of dead space between the pelvic inlet superiorly and the inferior border of the void created following PE. When this void continues through the perineum, the EPD is defined as the superior border of any reconstruction to close the perineal wound.

Various methods of pelvic filling have been used to reduce the EPD after PE, including omentoplasty, Bakri obstetric balloons, or breast implants [2]. The pelvic filling volume (PFV) is defined here as the volume of any reconstructive implant or flap deliberately placed within the EPD in order to fill the empty space created within the pelvis.

The PDS was measured using routine sterile wash with a bladder syringe prior to any resection within the true pelvis. The table was tilted head down in the Lloyd-Davies position so that the pelvic inlet and fluid level were both perpendicular to the floor. The EPD was measured in the same manner once the resectional phase of the PE was completed, and the perineum reconstructed to be watertight, preventing sterile wash from draining out.

Finally, the PFV was measured using Archimedes’ water-displacement principle. An omentoplasty was placed into the already wash-filled EPD, and the wash that was displaced was then aspirated with a bladder syringe until the fluid level was returned to the pelvic inlet.

Ratios to calculate volumetric changes were then performed:

- EPD/PDS = the degree of increase of the pelvic dead space following PE.
- PFV/EPD = the ratio of filling of the pelvic dead space remaining following PE.

## Results

**Fig. 1:**
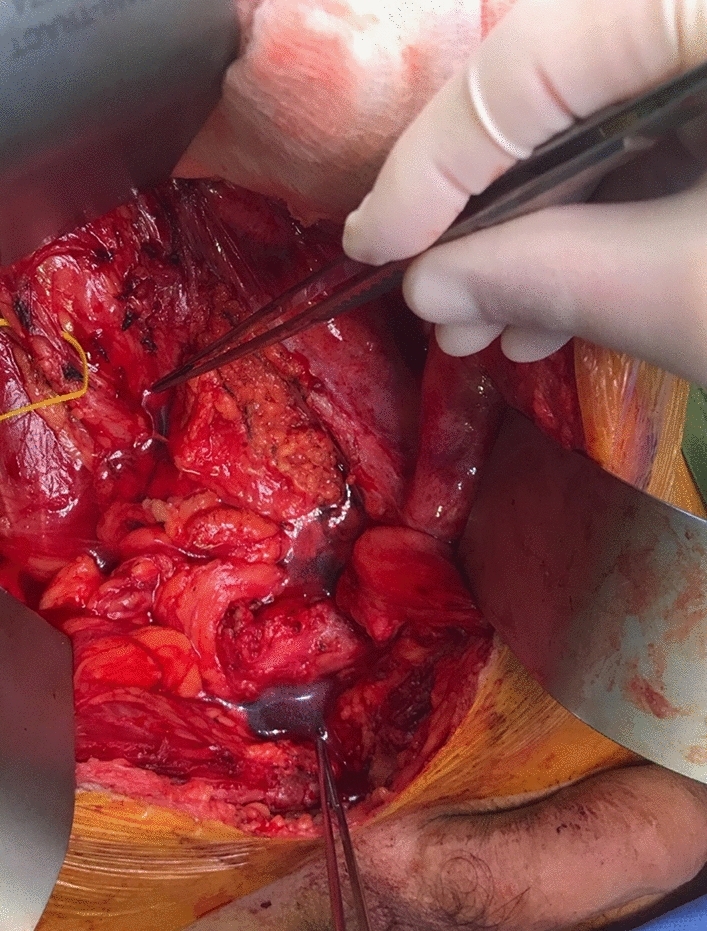
Measurement of the physiological pelvic dead space. Prior to any resection below the level of the pelvic inlet the patient was placed head down to render the pelvic inlet perpendicular to the floor. This space was then filled with sterile wash until the fluid level was at the pelvic inlet. Forceps mark the sacral promontory and the superior pubic symphysis with the fluid level seen between these points

**Fig. 2:**
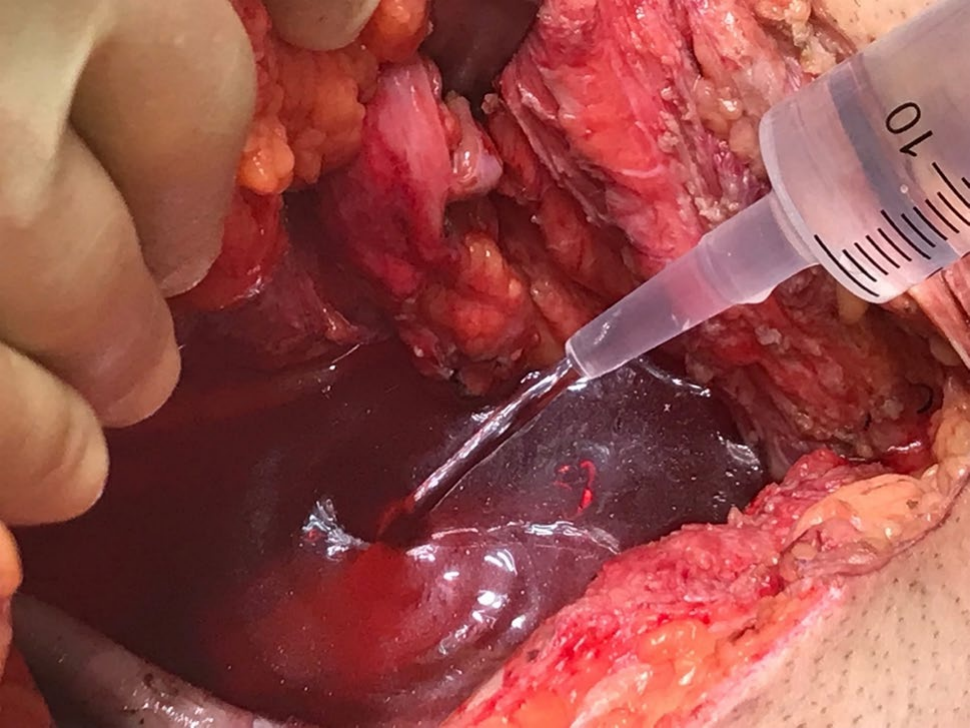
Measurement of the exenteration pelvic dead space. Following the resectional phase of pelvic exenteration a vertical rectus abdominis myocutaneous flap was sutured into the pelvic outlet to render it watertight. The table was again tilted head down and wash placed into the pelvis until the fluid level was at the pelvic inlet between the sacral promontory and the superior border of the pubic symphysis

**Fig. 3:**
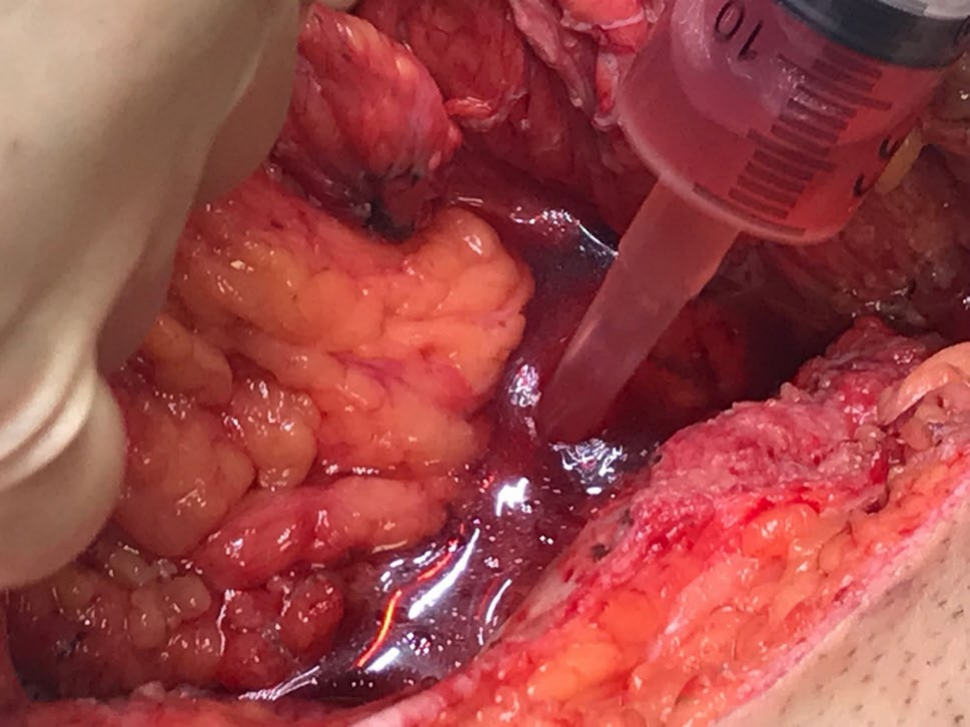
Measuring the pelvic filling volume. Immediately after measuring the exenteration pelvic dead space an omentoplasty that had already been mobilised was placed into the pelvis. The volume of the omentoplasty within the true pelvis was measured using Archimedes’ water displacement principle by aspirating wash until the fluid level was back to the level of the pelvic inlet

The methodology was trialled in a 50-year-old man with locally recurrent rectal cancer involving the right pelvic sidewall, coccygeus, and piriformis. He underwent total infralevator PE, lexicon coding was P3, A3, C2, left SV1, right SV3, right PM2, left PM1, vertical rectus abdominis myocutaneous flap (VRAM)/omentoplasty, and Wallace conduit [8]. Prior to any resection inferior to the pelvic inlet, the PDS volume was measured to be 50 ml (Fig. 1). Following the resectional phase, a VRAM was sutured into the perineal wound, rendering it watertight, and the EPD was measured at 540 ml (Fig. 2). An omentoplasty was mobilised into the EPD, displacing 130 ml of fluid to give the PFV (Fig. 3).

The ratio of PDS to EPD was calculated as 540 ml/50 ml demonstrating a 10.8-fold increase in dead space within the true pelvis. The PFV to EPD ratio was 130 ml/540 ml; therefore, the omentoplasty filled 24.1% of the dead space that it was placed into. The patient went on to develop an EPS complication with an infected pelvic fluid collection that required ultrasound guided-drainage prior to discharge.

## Discussion

Sterile solution and a bladder syringe have previously been employed to size appropriate breast implants for pelvic filling during reconstruction. However, this is the first instance in which changes in pelvic dead space have been objectively measured using defined anatomical landmarks [5]. The use of the PDS and EPD to define the magnitude of change in pelvic dead space, and the EPD and PFV to define the degree of filling of dead space, is likely to be more informative than the discrete measurements alone. Calculating ratios of measurements helps to manage anatomical variations between subjects [9]. VRAM flaps have been advocated for pelvic filling to mitigate EPS, as they are bulkier more voluminous flaps. This study demonstrates that even with a VRAM flap, the volume of dead space within the true pelvis above the neo-perineum increased by 10.8-fold, supporting the use of multiple techniques, including omentoplasty, to further reduce dead space.

There are practical limitations to the described methodology, including the additional time it adds to already lengthy procedures, and that the technique has inaccuracies which may lead to inconsistent measurements. This is illustrated in Fig. 3, where serous fluid and blood from the operative field are mixed with the wash in the syringe. It was assumed that the perineal wound was watertight; however, wash may have leaked inferiorly. In the case described the reconstructive strategy was planned preoperatively, and obtaining these measurements during surgery did not influence surgical decision-making. In high complexity PE with high sacrectomy, high subcortical sacrectomy, pubic bone resection, or tumour growth out of the pelvic inlet, there may be disruption of the bony landmarks, upon which this technique relies. The pathophysiology of EPS is poorly defined, and other factors may be relevant, including migration of the bowel into the pelvis, loss of mechanical support from the pelvic outlet, relative ischaemia secondary to radiotherapy, exposure of bony surfaces, scar tissue from previous surgery, and ligation of the internal iliac system [6]. Changes in the volumes of dead space may not be as important as these other factors, and it is notable that despite omentoplasty and a VRAM flap for pelvic filling in the presented case, an infected pelvic collection occurred.

The concept of loss of domain in the context of massive ventral hernias is another recently recognised pathological process where volumetric changes within the peritoneal cavity occur. Several studies have aimed to establish a volumetric definition for loss of domain through analysis of cross-sectional imaging; through an international Delphi study the Sabbagh volumetric definition was selected owing to its ease of application in a clinical setting using preoperative computed tomography (CT) [10]. Preoperative CT scans in 28 patients undergoing PE were analysed using automated CT segmentation using deep learning-driven software, which was able to predict EPD by subtracting organs and tissues resected during PE from the volume of the true pelvis [11]. The method described here could have value in validating radiological assessments and ultimately lead to a volumetric definition for EPS that correlates with adverse outcomes, thereby enhancing the understanding and management of this issue.

## Conclusions

As PE for more invasive tumours becomes increasingly feasible the prevalence and severity of EPS are likely to rise. A better understanding and quantification of the pathophysiological factors causing EPS is urgently required, with volumetrics representing a possible avenue of objective inquiry.

Research into EPS is challenging because of the multifactorial nature of this issue. This study demonstrates that the pathological volumetric changes relating to PE are objectively measurable. This preliminary work suggests a path toward a clinically helpful volumetric definition for EPS that could aid planning the requirements and means of pelvic filling in the reconstructive phase of PE.

## Data Availability

Datasets generated during the current study are available from the corresponding author on reasonable request.

## Abbreviations

CT: Computed tomography
EPD: Exenteration pelvic dead space
EPS: Empty pelvis syndrome
PDS: Physiological pelvic dead space
PE: Pelvic exenteration
PFV: Pelvic filling volume
VRAM: Vertical rectus abdominis myocutaneous flap
